# Degradation of Host Proteins and Apoptosis Induced by Foot-and-Mouth Disease Virus 3C Protease

**DOI:** 10.3390/pathogens10121566

**Published:** 2021-11-30

**Authors:** Jiamin Yi, Jiangling Peng, Jingjing Ren, Guoqiang Zhu, Yi Ru, Hong Tian, Dan Li, Haixue Zheng

**Affiliations:** State Key Laboratory of Veterinary Etiological Biology, National Foot and Mouth Disease Reference Laboratory, Lanzhou Veterinary Research Institute, Chinese Academy of Agricultural Sciences, Lanzhou 730046, China; yijm5599@163.com (J.Y.); 13592582178@163.com (J.P.); yashangzhuo@163.com (J.R.); Zhuguoqiang915@163.com (G.Z.); ruyi@caas.cn (Y.R.); tianhong@caas.cn (H.T.)

**Keywords:** FMDV, 3C protease, NF-κB signaling, apoptosis, CrmA

## Abstract

Foot-and-mouth disease (FMD), induced by the foot-and-mouth disease virus (FMDV), is a highly contagious disease of cloven-hoofed animals. Previous studies have reported that FMDV 3C protease could degrade multiple host proteins; however, the degradation mechanism mediated by FMDV 3C is still unclear. Here, we found that transient expression of FMDV 3C degraded various molecules in NF-κB signaling in a dose-dependent manner, and the proteolytic activity of FMDV 3C is important for inducing degradation. Additionally, 3C-overexpression was associated with the induction of apoptosis. In this study, we showed that an apoptosis inhibitor CrmA abolished the ability of 3C to degrade molecules in NF-κB signaling. Further experiments using specific caspase inhibitors confirmed the irrelevance of caspase3, caspase8, and caspase9 activity for degradation induced by 3C. Altogether, these results suggest that FMDV 3C induces the widespread degradation of host proteins through its proteolytic activity and that the apoptosis pathway might be an important strategy to mediate this process. Further exploration of the relationship between apoptosis and degradation induced by 3C could provide novel insights into the pathogenic mechanisms of FMDV.

## 1. Introduction

Foot-and-mouth disease (FMD) is an acute, febrile, highly contagious disease of cloven-hoofed animals caused by the foot-and-mouth disease virus (FMDV) [1]. FMDV, a member of the genus Aphthovirus in the *Picornaviridae* family, is a non-enveloped icosahedral virus. Its genome, a positive single-stranded RNA of approximately 8.5 kb, encodes a large polyprotein. Viral proteases process the polyprotein into four structural proteins (VP4, VP2, VP3, and VP1), eight nonstructural proteins (L, 2A, 2B, 2C, 3A, 3B, 3C, and 3D), and several intermediates [2].

3C protease, which exists in all picornaviruses, can cleave viral polyproteins and various host factors through its catalytic activity [3,4]. FMDV 3C has been proven to subvert host responses by cleaving multiple cellular proteins [3,4]. For instance, 3C cleaves histone 3 (H3), eukaryotic initiation factor 4AI (eIF4AI), and eukaryotic initiation factor 4GI (eIF4GI) to block host transcription and translation [5,6,7]. Src-associated in mitosis of 68 kD (Sam68), Ras GTPase-activating protein-binding protein 1 (G3BP1), and heterogeneous nuclear ribonucleoprotein K (hnRNP K) are cleaved by 3C to promote FMDV replication [8,9,10]. Moreover, FMDV 3C cleaves IKKγ to inhibit the innate immune response [11]. However, the specific mechanisms of 3C-induced degradation of some proteins such as γ-tubulin, Karyopherin 1 (KPNA1), double-stranded RNA-activated protein kinase (PKR), and autophagy-related 5 homolog–12 homolog (ATG5–ATG12) are still unclear [12,13,14,15]. Therefore, we attempted to demonstrate the mechanism of the extensive degradation induced by FMDV 3C.

Apoptosis is the process of programmed cell death that is selective and controlled [16,17]. Upon external or intrinsic stimuli, apoptosis is induced by the activation of a death signal and utilizes a cascade of caspases. The activation of caspases or other proteases induces multiple cleavage events, subsequently causing apoptosis-associated morphological changes, including membrane blebbing, chromatin condensation and cell shrinkage [16,17]. Previous studies have shown that 3C proteases of several picornaviruses can induce apoptosis to promote viral replication and subvert host immunity responses [4,17]; however, the modulation of apoptosis by FMDV 3C has not been reported.

The present study verified that FMDV 3C degrades multiple molecules involved in NF-κB signaling through its proteolytic activity. Moreover, 3C induces apoptosis. Further research found that apoptosis inducers caused the same molecules to degrade and an apoptosis inhibitor, CrmA restored the abundance of proteins degraded by 3C-expression, whereas the disruption of caspase3, caspase8, and caspase9 activity did not affect the degradation events. Thus, our study reveals a critical role of 3C in degrading various molecules involved in NF-κB signaling and inducing apoptosis and confirms that an apoptosis inhibitor impedes the degradation induced by 3C.

## 2. Materials and Methods

### 2.1. Cells and Viruses

Human embryonic kidney 293T (HEK293T) cells (ATCC; CRL-11268) and porcine kidney PK-15 cells (ATCC; CCL-33) were cultured at 37 °C with 5% CO_2_ in Dulbecco’s modified Eagle’s medium (DMEM, Gibco, Grand Island, NY, USA) supplemented with 10% fetal bovine serum (BI, Kibbutz Beit Haemek, Israel). The FMDV strain O/BY/CHA/2010 was isolated from a pig in China by our laboratory [18].

### 2.2. Plasmids

The coding sequence of FMDV 3C was fused with Flag-tag and cloned into the pCAGGS expression plasmid (pCAGGS-Flag-3C), which was constructed using standard molecular biology techniques. Various HA-tagged components including myeloid differentiation factor 88 (MyD88), tumor necrosis factor receptor-associated factor 6 (TRAF6), transforming growth factor-β-activated kinase 1 (TAK1), TAK1 binding proteins (TAB1, TAB2, and TAB3), and IκB kinase complex (IKKα, IKKβ, and IKKγ) expressing plasmids and CrmA used in this study were kindly provided by Professor Hongbing Shu (Wuhan University, Wuhan, China). The sequence identity of the plasmids was verified using DNA sequencing.

### 2.3. Antibodies and Reagents

Anti-β-actin mouse monoclonal antibody was purchased from Santa Cruz Biotechnology (Santa Cruz, CA, USA). Anti-Flag or anti-HA mouse monoclonal antibodies were purchased from Sigma-Aldrich (St. Louis, MO, USA). The goat anti-mouse IgG secondary antibody was purchased from Thermo Fisher Scientific (Waltham, MA, USA). Apoptosis Inducers Kit (C0005) was obtained from Beyotime (Shanghai, China).

### 2.4. Western Blotting

Western blotting was performed as described previously [19]. HEK293T cells were cultured in 12-well cell plates for 24 h and then transfected with the appropriate plasmids. Cells were collected at the indicated time points and lysed. Proteins were resolved by SDS-PAGE in acrylamide gels and transferred onto nitrocellulose membranes (Merck Millipore, Billerica, MA, USA). The membranes were incubated with 5% non-fat dry milk in TBS for 30 min at room temperature and probed with appropriate primary antibodies and dye-light fluorescent conjugated secondary antibodies. Antibody-antigen complexes were visualized using ECL detection reagents.

### 2.5. Site-Direct Mutagenesis

Mutations of FMDV 3C were introduced into the FMDV 3C-encoding plasmids using Pyrobest DNA Polymerase (TaKaRa, Tokyo, Japan) according to the manufacturer’s instructions. Site-directed mutagenesis primers containing nucleotide substitutions targeting amino acids H46, D84, and C163 were designed, leading to substitutions of H to Y, D to N, and C to G, respectively, which generated mutations in the catalytic triad of 3C, pCAGGS-Flag-3C(H46Y), pCAGGS-Flag-3C(D84N), and pCAGGS-Flag-3C(C163G), respectively [20]. A mutant of FMDV 3C with intact catalytic activity pCAGGS-Flag-3C(H205R) was generated using a similar method [20]. Correct nucleotide substitutions were confirmed using DNA sequencing.

### 2.6. Caspase Inhibitors Treatment

HEK293T cells were treated with dimethyl sulfoxide (DMSO) (Sigma-Aldrich, St. Louis, MO, USA), Z-DEVD-FMK (Apexbio Technology LLC, Houston, TX, USA), Z-IETD-FMK (Apexbio Technology LLC, Houston, TX, USA), and Z-LEHD-FMK (Selleck Chemicals, Houston, TX, USA) (20 mM) for 2 h, followed by co-transfection of various molecules in NF-κB signaling with 3C or empty vectors for 18 h. The samples were harvested and analyzed using western blotting.

### 2.7. Cytotoxicity Test

HEK293T cells were transfected with various plasmids for 18 h. Cytotoxicity tests were performed using the Cell Counting Kit-8 (CCK-8) (Biosharp, Hefei, China) according to the manufacturer’s protocol.

### 2.8. Quantitative Reverse-Transcription-PCR(RT-qPCR) Analysis

Total RNA was extracted using TRIzol reagent (TaKaRa, Tokyo, Japan). Transcription levels of *CASP3* and *CASP8* genes were normalized to the levels of *GAPDH* mRNA. The porcine gene-specific primer sequences were as follows: 5′-TCTTCAGAGGGGACTGCTGTA-3′ (forward) and 5′-CCTCGGCAGGCCTGAATTAT-3′ (reverse) for *CASP3*, 5′-CCAGGATTTGCCTCCGGTTA-3′ (forward) and 5′-CAGGCTCAGGAACTTGAGGG-3′ (reverse) for *CASP8*, 5′-ACATGGCCTCCAAGGAGTAAGA-3′ (forward) and 5′-GATCGAGTTGGGGCTGTGACT-3′ (reverse) for *GAPDH*.

Genomic copy numbers of FMDV were quantified using a TaqMan probe to conserved regions within FMDV *3D* [21]. The specific primer sequences were as follows: 5′-ACTGGGTTTTACAAACCTGTGA-3′ (forward) and 5′-GCGAGTCCTGCCACGGA-3′ (reverse). The probe sequence was 5′-TCCTTTGCACGCCGTGGGAC-3′, which was modified with a 5′ 6-carboxy-fluorescein (FAM) dye and a 3′ nonfluorescent quencher with tetramethylrhodamine (TAMRA).

### 2.9. Flow Cytometric Analysis of Apoptosis

HEK293T cells were transfected with the various plasmids for 48 h. The apoptosis occurrence was assessed by staining a cell population with AbFlour^TM^ 488 annexin V and propidium iodide (PI) using an Annexin V-AbFlour^TM^ 488 Apoptosis Detection Kit (Abbkine, Wuhan, China).

### 2.10. Caspases Activity Assay

HEK293T cells were treated with DMSO, Z-DEVD-FMK, Z-IETD-FMK, and Z-LEHD-FMK (20 mM) for 2 h. The culture medium was replaced after 2 h. Caspase activity was measured after 16 h using a Caspase 3 Activity Assay Kit, a Caspase 8 Activity Assay Kit, or a Caspase 9 Activity Assay Kit (Beyotime, Shanghai, China).

### 2.11. Statistical Analysis

All the data were analyzed using GraphPad Prism software (GraphPad Software Inc., La jolla, CA, USA). The significance of the results was assessed using an unpaired two-tailed Student’s *t*-test. The results are presented as mean values ± standard error of the mean (mean ± SEM) of three replicates. All experiments were repeated independently at least three times, and the data are shown as one representative experiment.

## 3. Results

### 3.1. FMDV 3C Degrades Various Molecules in NF-κB Signaling

**Figure 1:**
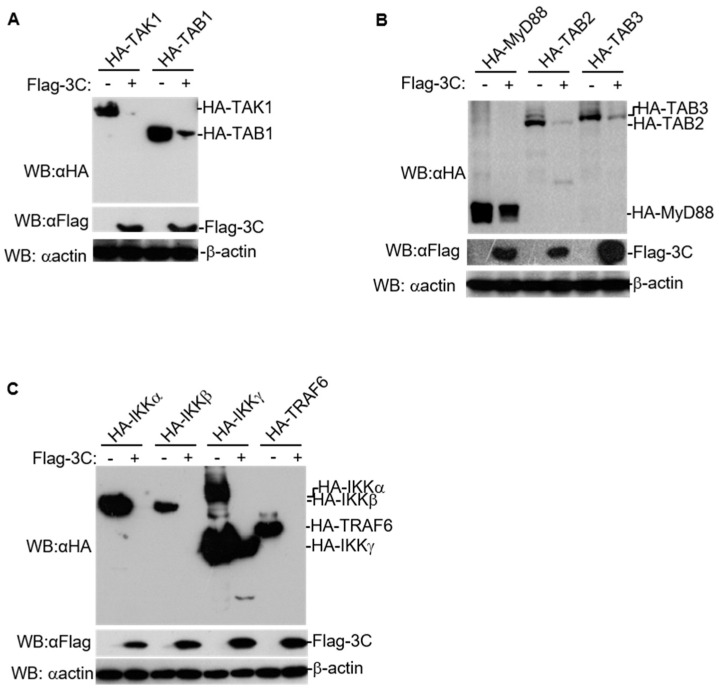
FMDV 3C degrades host proteins involved in NF-κB signaling. (**A**–**C**) HEK293T cells were transfected with empty vectors or Flag-3C-expressing plasmids (1 µg) together with indicated HA-tagged adaptors expressing plasmids (2 µg). After 24 h, total protein extracts were collected and analyzed by western blotting using antibodies specific to Flag, HA, or β-actin. WB, western blotting.

3C plays an important role in virus replication and subverting host responses during picornavirus infection [3,4]. To investigate the effect of FMDV 3C on the expression of molecules in NF-κB signaling, HEK293T cells were transfected with various expression plasmids of host proteins involved in NF-κB signaling (MyD88, TRAF6, TAK1, TAB1, TAB2, TAB3, IKKα, IKKβ, and IKKγ) combined with FMDV 3C or empty vectors. The expression of host proteins in NF-κB signaling and FMDV 3C was measured using western blotting. We observed that FMDV 3C substantially reduced the expression of these adaptors (Figure 1A–C). Interestingly, only the cleaved bands of TAB2 and IKKγ were detected in 3C expressing cells. There is also the possibility that the cleaved bands were separated from fused HA-tag or had a low abundance; thus, these bands could not be detected by anti-HA antibodies well. These results suggest that 3C induces the degradation of various host proteins involved in NF-κB signaling.

### 3.2. FMDV 3C Degrades Host Proteins Involved in NF-κB Signaling in a Dose-Dependent Manner

**Figure 2:**
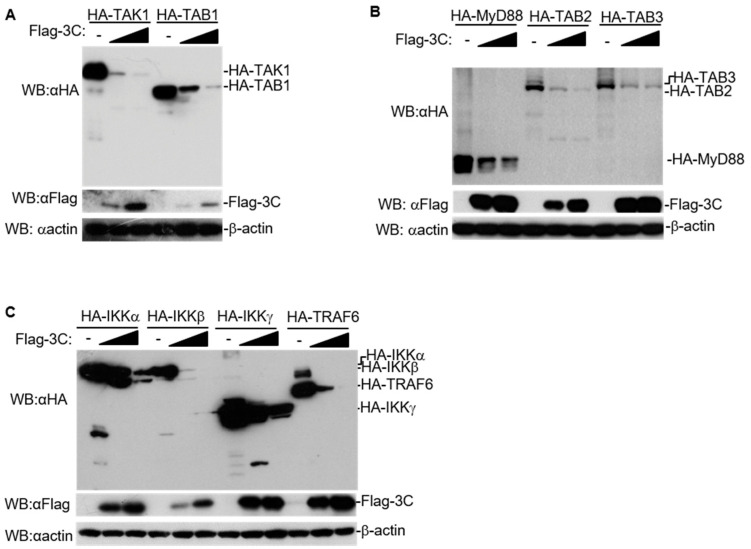
FMDV 3C degrades host proteins involved in NF-κB signaling in a dose-dependent manner. (**A**–**C**) Indicated HA-tagged adaptors expressing plasmids (2 μg) and increasing amounts of Flag-3C-expressing plasmids (0, 0.5, and 1.0 μg) were co-transfected into HEK293T cells. After 24 h, total protein extracts were collected and analyzed by western blotting using antibodies specific to Flag, HA, or β-actin. WB, western blotting.

To confirm the regulatory effect of 3C on the expression of these adaptors, we transfected HEK293T cells with various expression plasmids of host proteins involved in NF-κB signaling combined with increasing doses of FMDV 3C. The expression of these adaptors reduced with the increase in expression of 3C (Figure 2A–C). Moreover, with increasing dosages of 3C, cleavage products of TAB2, IKKα, IKKβ, and IKKγ were also degraded. This indicated that 3C had a substantial effect on the expression of host proteins involved in NF-κB signaling in a dose-dependent manner.

### 3.3. Proteolytic Activity Is Involved in the Degradation Induced by FMDV 3C

**Figure 3:**
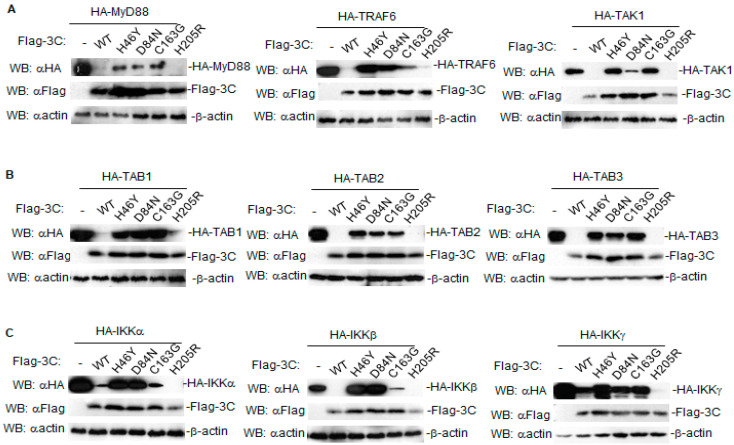
The protease activity of FMDV 3C is involved in the downregulation of host proteins involved in NF-κB signaling. (**A**–**C**) HEK293T cells were transfected with empty vectors, wild type 3C, or 3C mutants (H46Y, D84N, C163G, H205R) (1 μg) together with indicated adaptors (2 μg). After 24 h, total protein extracts were collected and analyzed by western blotting using antibodies specific to Flag, HA, or β-actin. WB, western blotting.

Previous studies have illustrated that the protease activity of 3C is essential for the modulation of host responses [5,6,7,8,9,11,12,13]. To determine whether the degradation of 3C is dependent on its proteolytic activity, we generated a series of mutants, including 3C(H46Y), 3C(D84N), and 3C(C163G) with disrupted catalytic activity as well as 3C(H205R) with intact catalytic [20]. The effect of different FMDV 3C mutants on the expression of host proteins involved in NF-κB signaling was investigated by western blotting. The results showed that 3C mutants with disrupted proteolytic activity had a weaker ability to decrease the abundance of host proteins, and even restored their expression levels entirely, while 3C(H205R) with intact catalytic activity induced degradation events similar to wild-type 3C, even more significantly (Figure 3A–C). Taken together, these results demonstrate that 3C degrades various host proteins, partly because of its proteolytic activity.

### 3.4. Expression of 3C Induces Apoptosis

**Figure 4:**
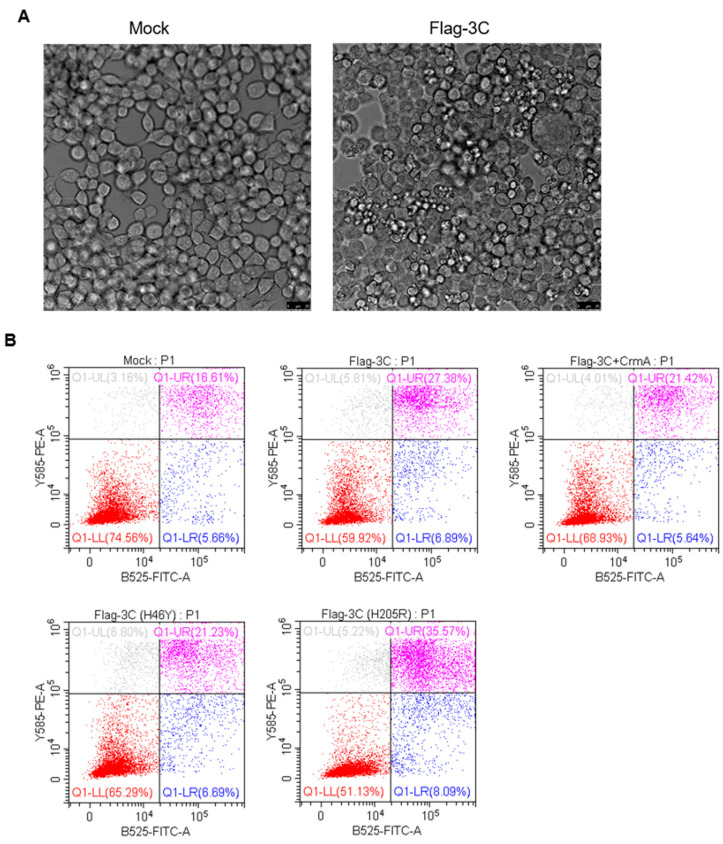

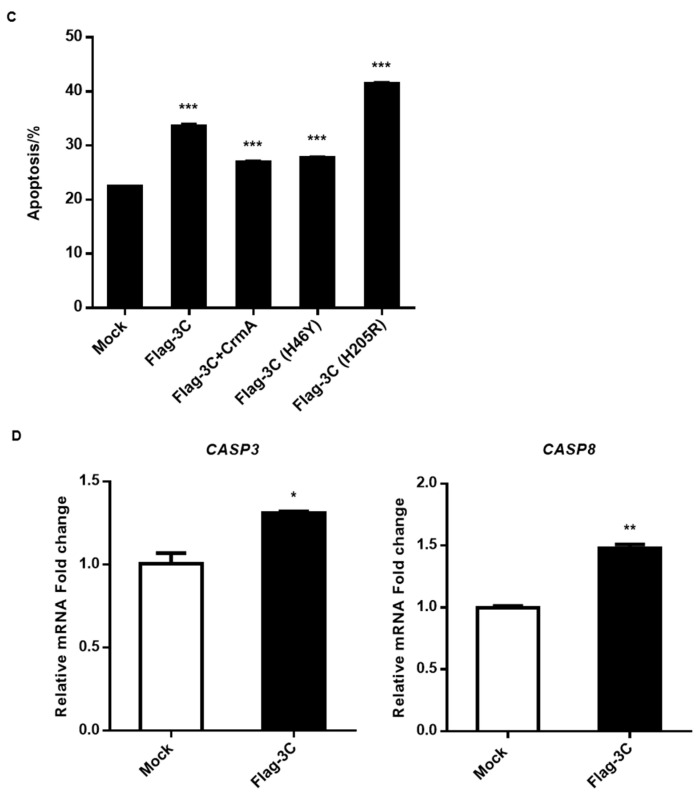
Expression of FMDV 3C induces cell apoptosis. (**A**) HEK293T cells were transfected with the indicated plasmids for 24 h or treated with apoptosis inducers for 12 h. Cells were visualized using a Laser scanning confocal microscope Leica TCS SP8 (Leica Microsystems, Wetzlar, Germany), 400× magnification. (**B**) A cell population with AbFlour^TM^ 488 annexin V and PI staining. HEK293T cells were transfected with the indicated plasmids for 48 h or treated with apoptosis inducers for 24 h. Cells were collected for analysis of the percentage of cells with apoptotic characteristics by flow cytometry. (**C**) Analysis of apoptosis occurrence in HEK293T cells from (**B**). (**D**) PK-15 cells were transfected with the indicated plasmids for 24 h. Expression levels of *CASP3* and *CASP8* were assessed by RT-qPCR. The results are presented as relative fold changes in mRNA levels compared to mock cells. The results represent the average of three replicates. Error bars represent SEM; *: *p* < 0.05; **: *p* < 0.01; ***: *p* < 0.001, compared to mock cells.

Previously, picornavirus 3C has been reported to control cell apoptotic pathways. For instance, 3C of poliovirus (PV) [22], enterovirus A71 (EV-A71) [23], Senecavirus A (SVA) [24,25], and coxsackievirus B3 (CVB3) [26], can trigger apoptosis. However, it remains unclear whether FMDV 3C affects apoptosis progression. In this study, we found that the expression of 3C led to characteristic apoptotic morphological changes, including cell shrinkage and plasma membrane blebbing (Figure 4A). 3C-expressing cells were subjected to flow cytometry to assess apoptosis, and the proportion of apoptotic cells among 3C-expression cells was markedly increased (Figure 4B,C). Moreover, we detected the mRNA levels of *CASP3* and *CASP8* in 3C-expressing cells. The results showed that 3C upregulated the expression of *CASP3* and *CASP8* in PK-15 cells (Figure 4D). Interestingly, expression of FMDV 3C induced apoptosis, regardless of whether the proteolytic activity is intact or disrupted, whereas 3C(H46Y) had a slighter effect on apoptosis compared to wild-type 3C and 3C(H205R) (Figure 4A–C). Collectively, 3C could induce apoptosis and the proteolytic activity of 3C might be involved in this process, although it is not essential.

### 3.5. CrmA Abolished the Ability of 3C to Degrade Host Proteins Involved in NF-κB Signaling

**Figure 5:**
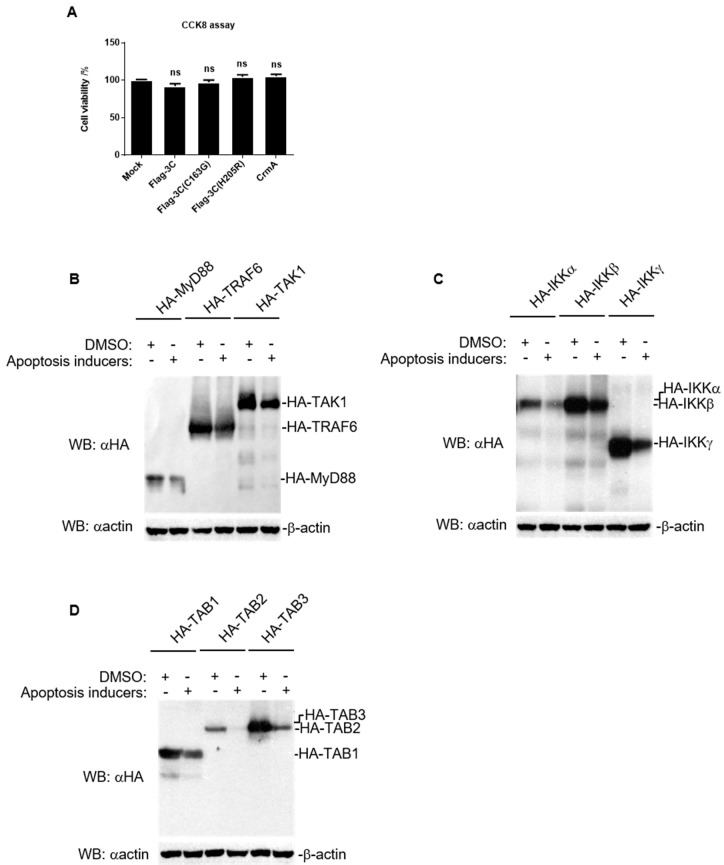
Apoptosis inducers cause similar degradation of host proteins involved in NF-κB signaling as that induced by FMDV 3C. (**A**) Cytotoxic effect in HEK293T cells transfected with the indicated plasmids were analyzed by CCK8 assay. The results represent the average of three replicates. Error bars represent SEM; ns, no significance (*p* > 0.05), compared to mock cells. (**B**–**D**) HEK293T cells were transfected with the indicated adaptors (2 μg) for 16 h and then were treated with apoptosis inducers for 6 h. Total protein extracts were collected and analyzed by western blotting with the indicated antibodies.

**Figure 6:**
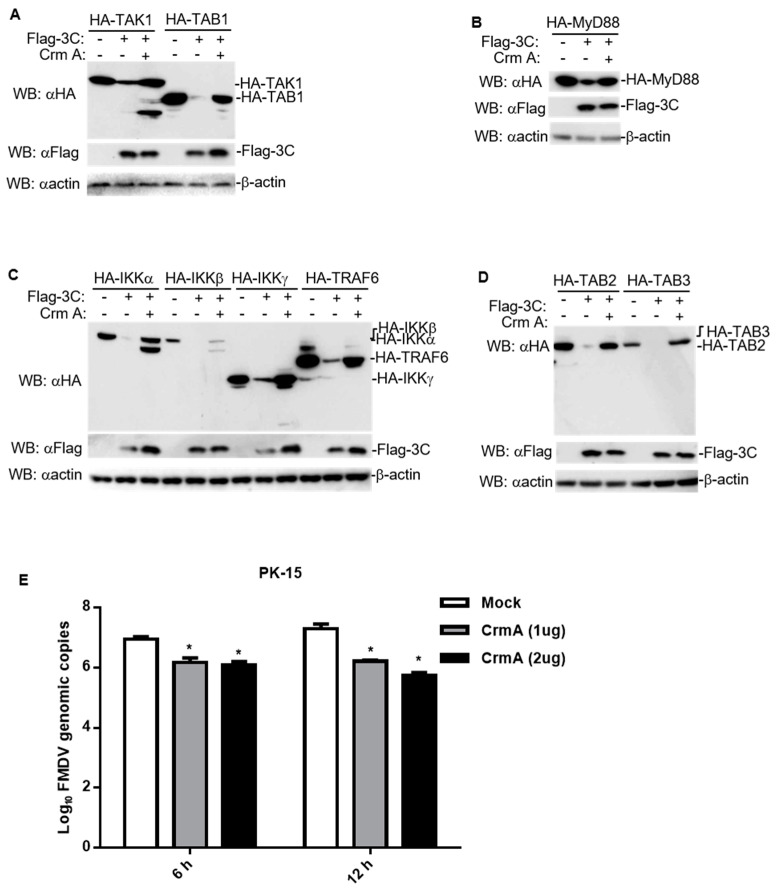
CrmA abolishes the ability of 3C to downregulate host proteins involved in NF-κB signaling and inhibits FMDV replication. (**A**–**D**) HEK293T cells were transfected with empty vectors or CrmA (1 μg) together with 3C (100 ng) and the indicated adaptors (2 μg). After 24 h, total protein extracts were collected and analyzed by western blotting using antibodies specific to Flag, HA, or β-actin. (**E**) PK-15 cells were transfected with empty vectors or CrmA in increasing dosage for 24 h and were infected with FMDV (6 or 12 h; MOI = 0.1). Genomic copy numbers of FMDV were quantified using the quantitative RT-qPCR assay. The results represent the average of three replicates. Error bars represent SEM; *: *p* < 0.05, compared to mock cells.

Since 3C triggers the occurrence of apoptosis, many proteases are activated and released during this process. We hypothesized that the apoptosis pathway might be involved in host protein degradation caused by 3C. To exclude any cytotoxic effect of 3C expression, we transfected HEK293T cells with FMDV 3C plasmids. As shown in the results, 3C did not decrease cell viability (Figure 5A), indicating that the decrease in host protein expression induced by 3C did not result from a cytotoxic effect. Interestingly, a similar marked decrease in the levels of host proteins involved in NF-κB signaling was also observed with apoptosis inducers treatment, supporting the hypothesis that apoptosis is involved in the degradation induced by 3C (Figure 5B–D). Here, we co-transfected CrmA, an apoptosis inhibitor (Figure 4B,C), with FMDV 3C and the indicated adaptors, and found that CrmA rescued the protein levels of the adaptors and the cleaved bands (e.g., TAK1, IKKα, IKKβ, and IKKγ) (Figure 6A–D). Moreover, the expression of CrmA also resulted in the inhibition of FMDV replication (Figure 6E). These results indicated that CrmA might be an effective inhibitor of 3C that is a virulence factor of FMDV. Moreover, apoptosis might be an important pathway by which 3C degrades multiple host proteins to facilitate FMDV replication.

### 3.6. Degradation of Host Proteins Involved in NF-κB Signaling Induced by FMDV 3C Is Independent of the Activity of Caspase3, Caspase8, and Caspase9

**Figure 7:**
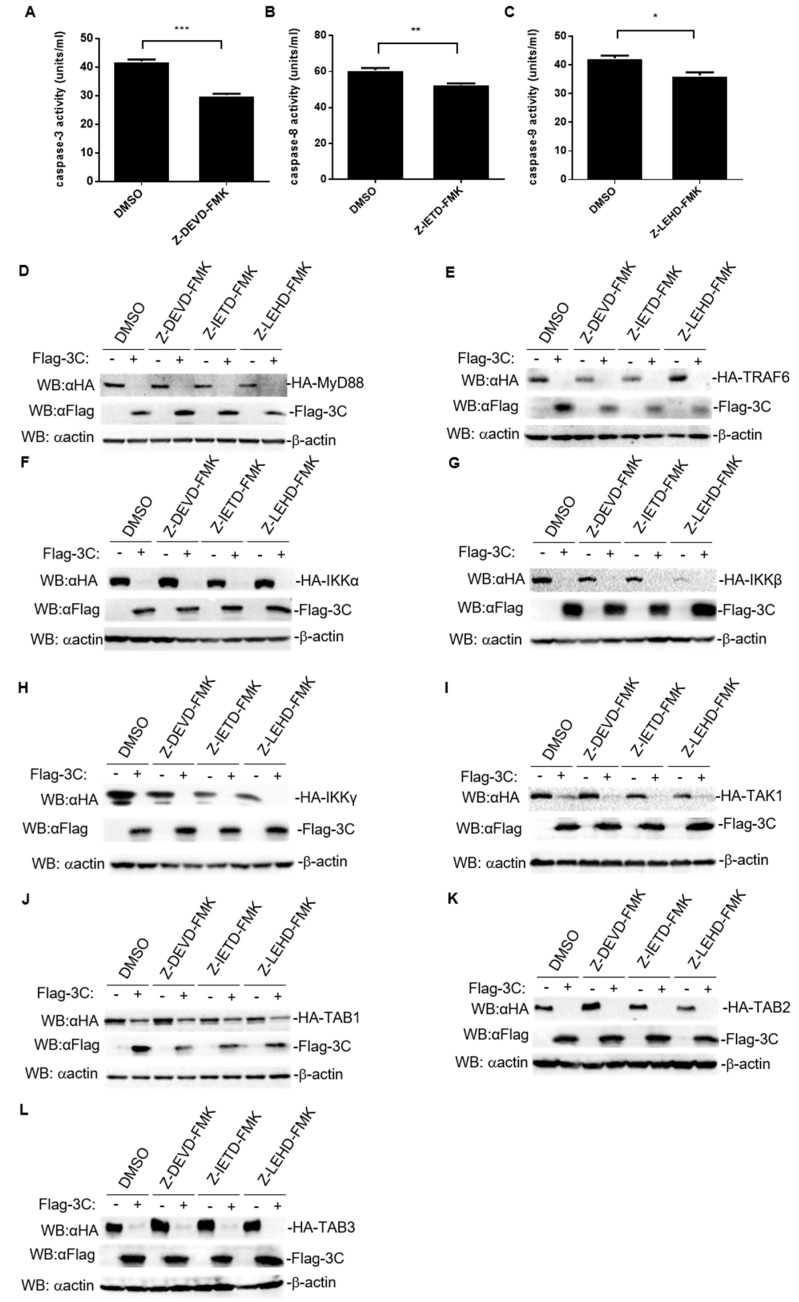
3C induces the degradation of host proteins involved in NF-κB signaling, independent of the activity of caspase3, caspase8, and caspase9. (**A**–**C**) Caspase activity was assessed after treatment with specific caspase inhibitors. The results represent the average of three replicates. Error bars represent SEM; *: *p* < 0.05; **: *p* < 0.01; ***: *p* < 0.001, compared to mock cells. (**D**–**L**) HEK293T cells (1 × 10^6^) were treated with DMSO, Z-DEVD-FMK, Z-IETD-FMK, or Z-LEHD-FMK (20 mM) for 2 h. Then, the indicated expression plasmids of host proteins (2 μg) and 3C or empty vectors (100 ng) were co-transfected for 24 h. Total protein extracts were collected and analyzed by western blotting using antibodies specific to Flag, HA, or β-actin.

Apoptosis is mediated by a series of highly complex cascade events that are triggered by apoptosis-inducing factors. During apoptosis signaling, caspase8 or caspase9 are activated to induce the activation of caspase3, which results in the occurrence of apoptosis [27]. To explore the specific apoptosis-associated molecules through which 3C targets the host proteins involved in NF-κB signaling, HEK293T cells were treated with three specific inhibitors: caspase3 inhibitor (Z-DEVD-FMK), caspase8 inhibitor (Z-IETD-FMK), and caspase9 inhibitor (Z-LEHD-FMK), which significantly decreased the activity of the caspases (Figure 7A–C). Unexpectedly, none of the inhibitors rescued the protein levels of host proteins involved in NF-κB signaling (Figure 7D–L). These data suggest that FMDV 3C degrades host proteins involved in NF-κB signaling, which is independent of the activity of caspase3, caspase8, and caspase9.

## 4. Discussion

It is well known that picornavirus 3C induces an abundance of cleavage events and degrades many host proteins [4]. Regarding cleavage events, they could be triggered by 3C itself or by the caspases activated by 3C. However, the degradation mechanism of FMDV 3C is still unclear.

Here, we found that FMDV 3C generally decreased the protein levels of several host proteins involved in NF-κB signaling in a dose-dependent manner (Figure 1A–C, Figure 2A–C). A previous study found that lysosomal degradation of PKR induced by FMDV 3C does not require protease activity of 3C [28]. Here, we found that mutations in the catalytic triad of 3C (H46Y, D84N, or C163G) partly abolished the degradation of host proteins involved in NF-κB signaling (Figure 3A–C), indicating that the protease activity of FMDV 3C is involved in the degradation of these host proteins.

Likewise, the host proteins degraded by FMDV 3C in this study have been shown to be the targets of other picornavirus 3C proteases. For instance, TAK1 could be cleaved by 3C of multiple enteroviruses (CVA16, CVA6, EV-A71, and EV-D68) [29,30], and TAB1, TAB2, and TAB3 could be cleaved by EV-A71 3C [30]. In addition, both hepatitis A virus (HAV) and FMDV 3C can cleave IKKγ to impair the production of interferons [11,31]. However, there are no host factors, which have been shown to mediate the degradation events in these studies. Hence, studies have shown that 3C is an effective factor for picornaviruses to modulate apoptosis. For instance, 3C proteases of PV [22], SVA [24,25], EV-A71 [23], and CVB3 [26], have been shown to be pro-apoptotic factors, while human rhinovirus (HRV) 3C suppresses apoptosis and induces non-necrotic cell death [32]. Here, we found that FMDV 3C is also an apoptosis inducer (Figure 4A–D), which might help 3C target more host factors and promote FMDV proliferation. We also showed that the induction of apoptosis caused the same substrates to degrade and the apoptosis inhibitor CrmA blocked 3C-induced degradation (Figure 5B–D, Figure 6A–D), suggesting that FMDV 3C might degrade host proteins involved in NF-κB signaling in an apoptosis-dependent pathway; however, caspase3, caspase8, and capsase9 activity are not essential (Figure 7D–L). Importantly, CrmA, a member of the serpin family encoded by the cowpox virus, was found to be an inhibitor of caspase1 and caspase8 which are key molecules in apoptosis [33]. CrmA has been shown to protect cells from apoptosis [34]. Caspase1, a cysteine protease that folds like a serine protease (like chymotrypsin), was originally described as a target of CrmA, their interaction was described as an example of a “cross-class inhibition” [35]. CrmA acts as a protease inhibitor and interacts with the protease through a cleavage site within it, which is a pseudosubstrate mechanism [36]. The FMDV 3C protease is a cysteine protease with a chymotrypsin-like fold. Therefore, it is possible that CrmA directly inhibited the proteolytic activity of FMDV 3C. However, CrmA had a stronger effect in impeding the degradation compared to mutants with mutations in the active sites of 3C (Figure 3A–C, Figure 6A–D). This suggests that apoptosis might be another pathway through which CrmA interrupts the degradation induced by FMDV 3C. In addition, the induction of apoptosis was not entirely dependent on the proteolytic activity of 3C (Figure 4B,C). Therefore, clearly dissecting the primary effects due to the proteolytic activity of the FMDV 3C protease and secondary effects resulting from 3C-induced apoptosis is challenging.

In summary, we identified the antagonistic role of FMDV 3C, as an apoptosis inducer, on the expression of various host proteins involved in NF-κB signaling, while CrmA abolished the inhibitory effect and inhibited FMDV replication. Furthermore, the degradation induced by FMDV 3C was independent of caspase3, caspase8, and caspase9 activity. These results suggest that 3C is an important factor in inhibiting innate immunity and promoting viral replication during FMDV infection. However, the conclusion that apoptosis is involved in the degradation induced by 3C is preliminary, and the specific mechanism of the inhibitory effect of CrmA on the degradation and apoptosis induced by 3C still needs to be studied. Clarifying these queries might help us understand the pathogenic mechanism of picornavirus and develop efficient 3C inhibitors to prevent picornavirus infection.

## Data Availability

Not applicable.
